# Reframing trauma recovery for public health: introducing the recovery assemblage framework

**DOI:** 10.3389/fpubh.2026.1868296

**Published:** 2026-09-03

**Authors:** Victoria Christodoulides, Maike Klein

**Affiliations:** 1 Department of Health, University of Bath, Bath, United Kingdom; 2 Division of Health Research, Lancaster University, Lancaster, United Kingdom

**Keywords:** assemblage, childhood trauma, community, health humanities, public mental health, social determinants of health, trauma recovery

## Abstract

Psychological trauma represents a major global health challenge, with adverse childhood experiences (ACEs) contributing to long-term difficulties in mental and physical health, relationships, and wellbeing. Despite this, dominant models of trauma recovery are largely grounded in biomedical and individualized paradigms, and prioritize linear pathways focused on symptom reduction and therapeutic outcomes. Such approaches risk locating responsibility for recovery within the individual, overlooking the broader social, relational, and structural conditions that shape how recovery is understood and practiced. In this paper, we introduce the Recovery Assemblage Framework for Trauma (RAF-T), a conceptual framework empirically informed by a feminist participatory action research study with adult survivors of childhood trauma conducted within UK community-based trauma recovery contexts. Drawing on feminist new materialism, posthuman ethics, and affective pedagogy, RAF-T reconceptualises recovery as dynamic, relational and contextually embedded, shaped through relations among bodies, lived experience, relationships, material conditions, environments, institutions, time, and wider social conditions. Rather than conceptualizing recovery through discrete domains, stages, principles, or resources, RAF-T facilitates reflection on how multiple influences become entangled and how their changing configurations may enable, constrain, or reshape possibilities for recovery within particular contexts. RAF-T therefore positions trauma recovery not solely as an individual or clinical endeavor, but as a population health concern shaped through communities, services, systems, and everyday environments. While grounded in this empirical context, RAF-T has potential relevance across wider trauma contexts; however, further research is needed to examine its applicability across different populations, cultural settings, and health systems. We discuss implications of RAF-T for public mental health, research and practice to facilitate a more relational, material, contextual, and systems-orientated understanding of trauma recovery. RAF-T offers a novel conceptual resource for exploring trauma recovery as a population health concern while remaining open to further empirical refinement and examination across different contexts.

## 1 Introduction

Adverse childhood experiences (ACEs), many of which have the potential to result in psychological trauma, are a major global public health concern, with substantial and enduring impacts on population mental health. The World Health Organization (1) estimates that approximately one billion children worldwide experience some form of violence or adversity each year, including abuse, neglect, and household dysfunction. These experiences are commonly conceptualized as adverse childhood experiences (ACEs), and are strongly associated with increased risk of mental health difficulties, including depression, anxiety, substance use, and suicidality, and poorer physical health outcomes across the life course (2–4). In England and Wales, the Crime Survey for England and Wales (CSEW) estimated that 13.6 million people aged 18 years and over (29.0%) had experienced emotional abuse, physical abuse, sexual abuse or neglect before the age of 18 years (5). These figures underscore childhood adversity as a widespread public health issue with substantial implications for population health.

Despite this, dominant models of trauma recovery remain grounded in individualized clinical paradigms that prioritize individual pathology and symptom reduction, diagnostic remission, and time-limited therapeutic outcomes, often overlooking the social, relational, and structural contexts that influence both recovery experiences and health outcomes. Increasingly, policy and research, including the WHO *World Mental Health Report* (2022) and subsequent WHO guidance (6), advocate approaches that recognize mental health and recovery as shaped by wider social and systemic conditions. This aligns with how trauma survivors frequently describe recovery as non-linear and ongoing, emerging through dynamic interactions across relational, material, institutional, and structural contexts. These include relationships with services, communities, engagement with environments, and the wider social inequalities that shape opportunities for recovery. However, despite growing recognition of these influences in policy and lived experience, they remain insufficiently theorized within conceptual public health frameworks of trauma recovery. Few conceptual frameworks explain how these interacting conditions co-produce recovery or offer a coherent way of understanding their significance for recovery at a population level. Consequently, there remains a significant conceptual gap between contemporary public mental health policy and the theoretical tools available to understand, design, and support trauma recovery across everyday settings.

Addressing this limitation requires moving beyond viewing recovery solely as the alleviation of symptoms toward understanding it as a process through which people come to know, negotiate, and reshape themselves and their relations with others, places, institutions, and the material world. In this paper, new materialism provides a way of understanding recovery as emerging through dynamic relations between people, matter, institutions, and wider social conditions. Here, *matter* refers to both human bodies and the more-than-human world, including environments, spaces, technologies, creative materials, and the physical world more broadly, all of which actively participate in shaping experience rather than serving as passive backdrops (7). A pedagogical perspective focuses on how people learn, make meaning, and transform through everyday encounters, relationships, and lived experience. Together, these perspectives shift attention beyond treatment toward the broader conditions through which people adapt, reconstruct meaningful lives, and experience health and wellbeing within their everyday social and material worlds.

To address this conceptual gap, we introduce the Recovery Assemblage Framework for Trauma (RAF-T), which reconceptualises recovery as dynamic, relational, and contextually embedded while providing a conceptual lens through which its wider public health significance can be explored. The framework is informed by empirical insights from a feminist participatory action research study with adult survivors of childhood trauma in the UK, where knowledge was co-produced with experts-by-experience. Drawing on feminist new materialism, posthuman ethics, and affective pedagogy, RAF-T conceptualizes recovery as an ongoing process of relational *becoming* that unfolds through situated pedagogical encounters, relations and everyday experiences. Within RAF-T, recovery is understood to emerge through assemblages: dynamic configurations of bodies, memories, environments, institutions, and socio-cultural practices that co-constitute possibilities for healing and transformation (7, 8). Grounded in knowledge co-produced with experts by experience, RAF-T emerged through an iterative process in which empirical insights informed and refined its conceptual and theoretical development, bridging trauma theory, public health practice, and policy. This article therefore develops RAF-T, as a conceptual framework for exploring the entangled conditions that shape possibilities for trauma recovery, and considers its implications for public mental health, research, and practice.

## 2 Theoretical shifts in trauma recovery

The development of knowledge and practice around trauma recovery has emerged through a complex interplay of psychological, biomedical, and sociocultural traditions. Since Freud's early work on hysteria and psychic injury, trauma has largely been theorized within Western contexts through individualized frameworks that locate distress primarily within the psyche or body of the individual (9). Post-Freudian scholarship has broadly evolved this understanding across four overlapping domains: psychoanalytic, psycho-scientific, neurobiological, and sociocultural approaches (10). Psychoanalytic traditions, including talk therapies and relational approaches, have typically framed recovery as a process of integrating traumatic memory through narrative, meaning-making, and reflective engagement (11).

While highly influential, these approaches have been critiqued for sometimes abstracting trauma from the material and social conditions in which it occurs, and for assuming that articulation alone can produce recovery (12, 13). Psycho-scientific and cognitive-behavioral paradigms later gained prominence for their emphasis on measurement, diagnosis, and behavioral modification (14). These models conceptualize trauma responses as maladaptive patterns requiring correction or regulation, often privileging symptom reduction as the primary indicator of recovery (15). Although cognitive-behavioral therapies have demonstrated effectiveness in certain contexts, they have also been critiqued for having limited applicability across the full range of trauma types and insufficient attention to relational and contextual dimensions of distress (16, 17). Neurobiological and epigenetic research has further advanced understandings of trauma's physiological impacts, demonstrating how trauma can influence brain function, stress regulation, and gene expression (18, 19). Central to this work is the hypothalamic–pituitary–adrenal axis, which mediates stress responses and can become dysregulated following chronic trauma exposure (20, 21), and the fight/flight/freeze responses of the autonomic nervous system triggered by the status of the amygdala, thalamus, and frontal lobe (22).

While these insights have contributed to more compassionate, biologically informed understandings of trauma, they can also risk re-medicalising recovery by prioritizing internal regulation while marginalizing relational, pedagogical, and social factors. Sociocultural approaches, in contrast, have emphasized trauma's entanglement with broader systemic forces, including housing, education, healthcare, justice systems, war, and public policy (23). Within this perspective, trauma is understood as both individually experienced and socially situated, with recovery involving opportunities to relearn practices of connection, meaning-making, and mutual care (9, 24). Taken together, these traditions show how trauma recovery has been shaped by disciplinary boundaries prioritizing treatment, regulation, and measurable outcomes.

Efforts to expand trauma recovery theory have produced influential integrative frameworks, most notably the biopsychosocial model of health and illness (25) and Judith Herman's (9) phase-based approach. The biopsychosocial model, although not trauma-specific, integrates biological, psychological, and social dimensions of health, offering a holistic orientation that recognizes contextual contributors to distress. However, in practice, applications often remain outcome-oriented, emphasizing symptom management and functional restoration while paying limited attention to the relational and experiential dimensions of recovery (26). Herman's three-stage model, safety and stabilization, remembrance and mourning, and reconnection, marked an important shift toward understanding recovery as rooted in relationships and the reworking of personal meaning. However, its staged structure has been critiqued for failing to capture the non-linear and materially embedded realities of lived recovery (27, 28). Across both frameworks, the pedagogical aspects of recovery, meaning how individuals learn, unlearn, and renegotiate relationships, identities, and environments following trauma, remain comparatively underdeveloped.

Another influential framework emerging from positive psychology is the post-traumatic growth (PTG) model (29), which suggests that individuals may experience positive psychological change following trauma, including shifts in meaning, identity, and relationships. While PTG highlights the possibility of transformation after adversity, it has been critiqued for focusing primarily on individual cognitive processes and interpersonal relationships, often overlooking the broader material, structural, and ecological conditions that shape recovery trajectories and for privileging human-to-human relational change (30). More recent resilience research has increasingly recognized resilience as dynamic and context-dependent, arising through interactions between individuals and the social and ecological resources available to them, rather than as an individual trait or capacity (31). However, these approaches tend to focus on the factors and resources associated with adaptation to adversity, rather than how relational, material, temporal, embodied, ethical, and structural influences become entangled to shape changing possibilities for recovery.

Trauma-informed care (TIC) extends these developments at an organizational level. Grounded in principles proposed by the Substance Abuse and Mental Health Services Administration (32), including safety, trustworthiness, collaboration, empowerment, and cultural responsiveness, TIC seeks to reshape service cultures by recognizing the impact and prevalence of trauma. Although widely adopted (33, 34), recent reviews have highlighted significant variability in definition, implementation, and evaluation of TIC, alongside calls for conceptual frameworks and the need to understand the underlying processes or mechanisms of change (35, 36). Without deeper theoretical grounding, it risks becoming proceduralised, reduced to compliance-driven checklists that insufficiently engage with the relational and embodied dimensions of healing.

A comparable shift has occurred in addiction recovery science through the development of recovery capital theory. Originally developed by Cloud and Granfield (37), recovery capital theory holds that recovery is shaped not solely by individual motivation or clinical intervention, but by the breadth and depth of resources available to a person. These resources operate across three interrelated domains: personal capital (e.g., skills, health, resilience, self-efficacy), social capital (e.g., supportive relationships, family, peer networks), and community capital (e.g., access to housing, employment, education, and recovery-supportive environments). From this perspective, recovery is not simply the cessation of substance use, but the accumulation and mobilization of assets that enable individuals to build stable and meaningful lives (38). Ashford and Brown's (39) work further reframed recovery as a fundamentally emancipatory, strengths-based process grounded in lived experience, reinforcing an understanding of recovery as socially situated and shaped by access to resources, relationships, and opportunities.

Collectively, these frameworks have contributed to recent shifts in national drug policy in the UK (40), including the expansion of lived-experience recovery organizations (LEROs) as key providers of support. Typically staffed predominantly by people with lived experience, these organizations move recovery provision beyond traditional clinical healthcare settings into community-based environments. Unlike many healthcare services, LEROs often operate without strict intake thresholds; individuals can access support without first achieving abstinence or completing detoxification, aligning more closely with engagement-focused approaches such as the model of assertive linkage (41). Their flexible structure enables support across stages of recovery while countering stigma and social exclusion (42). Some LEROs adopt social enterprise models, producing goods or services that contribute to local economies and communities (43). Such initiatives position people in recovery as active contributors rather than passive recipients of care, fostering agency and community connection.

These developments resonate with critical feminist scholarship, which challenges the individualization of trauma. Burstow (27, 44) argues that trauma must be understood as rooted in structural violence and proposes trauma work as radical adult education. Extending this critique to contemporary trauma-informed approaches, Tseris (45, 46) and others highlight how trauma-informed practice can reproduce psychiatric authority when power relations remain unexamined. Drawing on decolonial and intersectional feminist scholarship, recovery is framed as inseparable from safety, justice, and collective change with community care, cultural resources, and resistance/advocacy positioned as integral to healing rather than supplementary (47, 48). Feminist participatory approaches further this critique by conceptualizing recovery as relational, affective, and co-produced, emphasizing transformation through embodied, social, and material practices that occur beyond clinical spaces, rather than individual symptom resolution (49–51).

Taken together, these traditions have progressively expanded understandings of trauma recovery beyond symptom reduction toward relational, contextual, and transformative accounts of recovery. However, they often remain shaped by the conceptual boundaries of the disciplines from which they emerge, emphasizing particular dimensions of recovery (e.g., cognition, biology, relationships, or resources) while leaving less space to consider how multiple influences become dynamically entangled across bodies, environments, institutions, and everyday practices. Recent reviews similarly critique dominant trauma frameworks for privileging biomedical and individualized models, and for insufficiently accounting for the sociocultural, relational, and systemic conditions that shape recovery (52). This points to a need for conceptual approaches capable of holding these influences in relation while remaining responsive to the lived, shifting realities of recovery. This paper does not seek to provide a comprehensive review or critique of existing trauma recovery frameworks, but to introduce RAF-T and position it in relation to these key approaches. RAF-T builds upon their contributions but differs in both its conceptualization and intended use. Rather than describing recovery through domains, stages, principles, protective factors, or resources, RAF-T conceptualizes recovery as continually emerging through dynamic relations among bodies, matter, environments, institutions, time, ethics, and lived experience.

Drawing on feminist new materialism, posthuman ethics, and affective pedagogy, the framework extends attention beyond identifying *what* influences recovery to exploring *how* these influences become entangled and what their changing configurations may enable, constrain, or make possible within particular contexts. In doing so, RAF-T is intended not as a prescriptive model or assessment framework, but as a heuristic resource that supports survivors, practitioners, researchers, and policymakers in identifying and reflecting upon these influences, their relations, and the possibilities they may create for recovery. As an initial conceptual articulation, it remains intentionally open to further empirical refinement, collaborative development, and theoretical examination.

## 3 Recovery assemblage framework for trauma (RAF-T)

The framework presented in this paper emerged from a larger feminist participatory action research project with adult survivors of childhood trauma conducted within UK community-based trauma recovery contexts. The project combined arts-based inquiry, collaborative analysis, and theoretical development to explore how trauma recovery might be understood and enacted beyond biomedical paradigms. While empirically grounded in this UK context, RAF-T is proposed as a conceptual framework with potential relevance beyond this setting, whose applicability across different health systems, cultural contexts, and populations requires further exploration. Before we describe the framework in more detail, we first provide some context on the model's empirical and theoretical grounding.

### 3.1 Empirical & theoretical grounding

The project involved 10 participants (henceforth, co-researchers) with lived experience of childhood trauma who participated in regular research assemblages, comprising workshops, Indeemo^1^ reflections, exhibition interactions, creative artifacts, and reflexive field notes. The project adopted a post-qualitative orientation informed by feminist new materialism, posthuman ethics, and affective pedagogy. Rather than seeking to reduce experience into stable themes or categories, post-qualitative inquiry understands knowledge as relational, emergent, and continually produced through relations among people, matter, practices, and contexts (53, 54). Analysis therefore involved iterative engagement across the research assemblages, attending to recurring patterns, affective intensities, material encounters, and moments of significance across creative practices, dialogue, embodied experiences, and researcher reflexivity. Interpreted through the project's theoretical framework, these relational moments informed the conceptual development of the RAF-T dimensions.

Feminist new materialism understands matter–including human bodies and the more-than-human world–as active in shaping experience rather than passive to human action (7). From this perspective, trauma and recovery are therefore not located solely within individuals but emerge through ongoing relations among bodies, histories, environments, and practices. Rather than viewing trauma as an internal condition residing within a person, feminist new materialism understands both trauma and recovery as produced through dynamic *entanglements*—the inseparable and mutually constitutive relations between people, matter, institutions, and practices through which experience emerges (7). Barad's (7) concept of *intra-action* extends this argument by proposing that entities do not pre-exist their relations; rather, they come into being through them. Trauma recovery, therefore, is understood not as repairing an isolated individual but as an ongoing reconfiguration of the relations among bodies, matter, institutions, histories, and practices through which recovery becomes possible.

Barad's notion of *spacetimemattering*, which understands space, time, and matter as mutually constituted rather than independent dimensions, further challenges linear models of recovery as movement from past harm toward future health. Instead, recovery involves learning new ways of living with ongoing entanglements, expanding the ways time, space, and embodiment are inhabited, rather than seeking closure or resolution. New materialist approaches also invite thinking in terms of *assemblages* rather than isolated individuals. Assemblages are dynamic constellations of people, ideas, practices, actions, and environments that mutually affect one another (8). This perspective treats all *matter* (human and more-than-human world) as active participants in meaning-making (55), shifting attention from what entities *are* to what they *do*: how they move, connect, and shape lived worlds (56). Assemblages remain fluid, with elements continually entering or leaving, thereby altering possibilities for feeling, acting, and relating (57). In the context of trauma, recovery is therefore shaped not only by therapy or diagnosis but by evolving configurations of relations, spaces, routines, practices, and material conditions (58). Thinking through recovery as an assemblage enables practitioners, policymakers, and people with lived experience to consider how recovery is continually shaped by changing relations among people, environments, services, material conditions, and wider social structures, rather than attributing progress or difficulty solely to the individual.

Secondly, *posthuman ethics* adds an explicitly political and relational perspective, conceptualizing subjectivity as an emergent practice shaped through entanglements among social and political structures, and the more-than-human world (59). Here, *more-than-human* refers to the wider material world—including environments, technologies, spaces, and other non-human forms of matter—that actively participate in shaping experience alongside social and political structures. Social and political formations, including gender, class, race, and (dis)ability, are understood as intersecting with these material conditions to shape possibilities for health, recovery, and becoming. Recovery, from this perspective, is understood through a collective ethical orientation grounded in recognizing interdependence and cultivating compassion, responsibility, and reciprocity across human and more-than-human relations. This aligns with feminist and survivor-led critiques that situate trauma within broader structures of power and oppression rather than solely within individual psyches (27, 50).

Lastly, *affective pedagogy* (51), understands recovery as learning through feeling and doing rather than solely through reflection or dialogue. Here affect is not limited to identifiable emotions or feelings but refers more broadly to relational intensities and shifts in capacities that emerge through encounters among bodies, matter, environments, and wider social and historical conditions. Drawing on Spinoza's (60) understanding of the capacity to affect and be affected, these encounters may expand, constrain, or otherwise reshape capacities for feeling, acting, relating, and becoming. Within the larger FPAR study, arts-based practices (e.g., body mapping, thread work, clay, animation) functioned as pedagogical encounters that generated what Hickey-Moody (51) terms affective intensities: moments in which co-researchers sensed themselves and others differently, opening space for new meanings and capacities. The co-researchers described these creative practices as enabling them to relate to their bodies, relationships, and futures in ways not available within conventional treatment settings. Recovery knowledge thus emerges not only in clinical contexts but through everyday acts of doing, making, and relating. Affective pedagogy names these encounters as sites of learning and transformation, where learning emerges through embodied, relational, and affective experience rather than through formal instruction alone. Taken together, these theoretical perspectives provided the conceptual lens through which recurring patterns across the participatory inquiry were interpreted, ultimately informing the development of RAF-T as a heuristic framework for understanding trauma recovery as relational, material, affective, and continually emergent. The following section outlines how these theoretical and empirical insights were synthesized into the eight relationally entangled dimensions of RAF-T.

### 3.2 The framework

**Figure 1:**
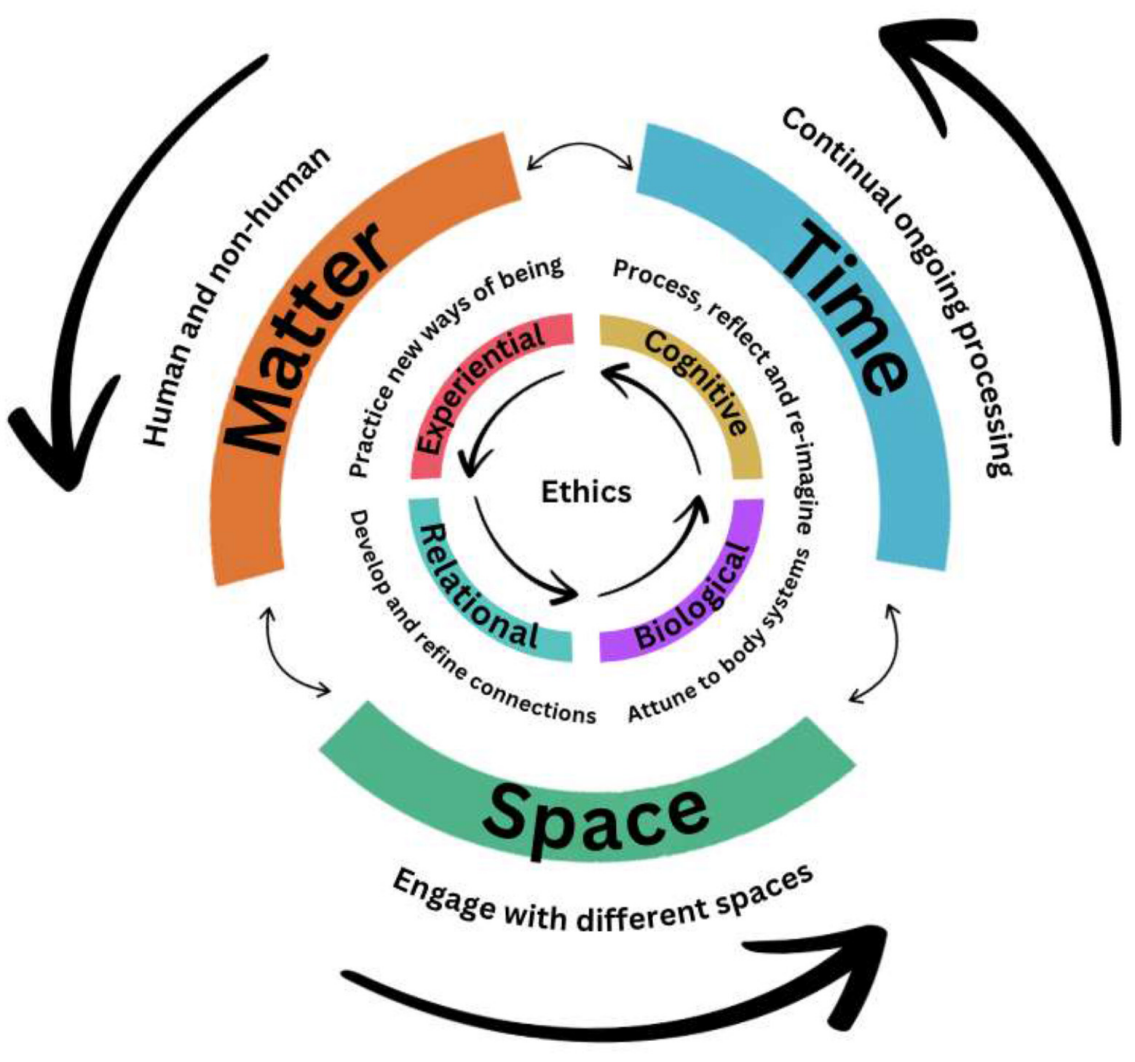
The Recovery Assemblage Framework for Trauma (RAF-T). Figure 1 depicts the RAF-T framework as two visually interconnected rings, organized in this way for conceptual clarity rather than to represent discrete, hierarchical, or nested levels of recovery. Matter, Time, and Space foreground material, temporal, and spatial dimensions of recovery, while Experiential, Cognitive, Relational, and Biological foreground experiential, interpretive, relational, and embodied dimensions. These distinctions are heuristic rather than bounded: all dimensions are understood as mutually constitutive, relationally entangled, and continually intra-acting across the assemblage. Ethics is positioned at the center and across the framework, highlighting how considerations of power, safety, reciprocity, and justice permeate all dimensions of recovery. Bidirectional arrows illustrate reciprocal and iterative relations but do not indicate interactions limited to adjacent dimensions. RAF-T therefore directs attention to how different configurations of these dimensions may expand, constrain, or reconfigure possibilities for recovery over time, rather than framing recovery as an individualized or linear trajectory.

The RAF-T framework condenses these insights into eight relationally entangled dimensions of recovery: matter, time, space, relationality, cognition, experiential, biological, and ethical (see Figure 1). These dimensions represent theoretically informed, interpretive conceptualisations of recurring entanglements that emerged through the iterative post-qualitative and participatory inquiry, rather than themes generated through conventional qualitative coding or predefined categories. RAF-T was not an intended outcome of the original study and is presented as an initial conceptual articulation, intended to support ongoing theoretical refinement, empirical examination, and collaborative development rather than as a definitive taxonomy of recovery.

These dimensions were not explicitly identified or named by co-researchers as bounded constructs but surfaced through repeated, often overlapping expressions of what participants described, created, and enacted as significant within their recovery experiences. Co-researchers engaged with these influences through body mapping, digital elicitation reflections (including photo, video, audio, and text uploads), thread work, creative workshops, group dialogue, and the collaborative curation of a public exhibition. These iterative encounters supported ongoing dialogue, collective reflection, and meaning-making, through which co-researchers continually shaped the knowledge from which RAF-T emerged. The dimensions are therefore heuristic, relational, and intentionally porous, offering conceptual lenses through which recovery can be explored rather than fixed or exhaustive categories.

Although presented separately for analytic clarity, the dimensions are not intended to function as discrete or independent categories. Consistent with RAF-T's post-qualitative and feminist new materialist orientation, each dimension foregrounds a particular aspect of recovery while remaining continually entangled with the others.

Material engagement, relational dynamics, temporal disruption, and affective intensities, for example, recurred across artifacts and encounters, illustrating how dimensions may become differently configured within particular recovery contexts. Rather than isolating components of recovery, RAF-T traces how dimensions intra-act^2^ to shape possibilities for knowing–doing–being. Their spatial separation within Figure 1 therefore supports conceptual legibility rather than implying discrete, hierarchical, or nested domains. The following section explores the significance of each dimension with reference to the empirical study.

#### 3.2.1 Matter

The matter dimension refers to the material dimensions of recovery, including bodies, textures, matter and environments. In the study, matter was active rather than neutral: clay absorbed anger, thread work cultivated patience and fragility, and body maps became surfaces for expressing scars, memories, and repair. One co-researcher's stitched doll, with visible seams and an embedded mirror beneath her locks of hair, materialized both fragmentation and the ongoing work of re-assembly, inviting reflection on what is seen, concealed, and carried in recovery. Materials did not simply represent experience but participated in shaping how it could be encountered, held, and reworked. As one co-researcher described, “…*I just worked intuitively… I feel like there is something of me in the pinch pot… something organic and that has a truth,”* illustrating how engagement with matter enabled forms of recognition not previously articulated. This dimension invites attention to how materials and embodied practices, such as creative media, movement, medication, or everyday matter can support or constrain recovery. For example, walking was described as shifting internal states, while environments such as a “*messy”* kitchen or a “tsunami of other tasks” could inhibit reflection. Meaning often emerged through doing rather than preceding it, enabling forms of expression beyond language. At times, this was also experienced through absence, where participants described states of disconnection in which “*you switch off… the absence of matter… sometimes feels like something,”* highlighting how both material engagement and its withdrawal shape recovery. This perspective resonates with van der Kolk's (22) view of trauma as embodied and accessed through sensory processes, while extending it by positioning material practices as active participants in meaning-making (7, 55).

#### 3.2.2 Experiential

The experiential dimension refers to how recovery is lived and sensed in everyday life, including bodily memories, affective intensities, and pre-verbal responses that often precede or exceed language. Co-researchers frequently described trauma as “*carried in the body*”, emerging not only through reflection but through doing. For example, one co-researcher noticed anger arising in the moment of engaging with clay: “*I am feeling a little angry now…why's that, and where is that coming from?*', highlighting how awareness was generated through embodied interaction rather than prior insight. These experiences extended beyond workshop settings into everyday encounters, where engagement with materials and environments supported the noticing of sensations, emotions, and meanings as they unfolded. As one co-researcher reflected, working with wet clay “*felt good to get messy… just to let things flow,”* illustrating a shift from control toward sensory engagement. Such moments created space for forms of awareness and response not always accessible through language. Across both shared and independent activities, co-researchers experimented with different embodied ways of responding, suggesting that recovery is shaped through everyday routines, environments, and micro-experiences. This perspective resonates with trauma scholarship emphasizing the persistence of somatic and pre-verbal memory (Rothschild, 2017), while affective pedagogy further highlights how such experiences emerge relationally between bodies and environments (51).

#### 3.2.3 Cognitive

The cognitive dimension resonates with cognitive and narrative approaches that emphasize the interpretation and reworking of traumatic experience (9, 15), while positioning cognition as one strand within a broader relational, embodied, and environmental assemblage. It extends beyond rational thought to include reflection, meaning-making, and the ongoing negotiation of how experiences are understood, narrated, or resisted. Within the study, sense-making emerged through distributed and situated encounters rather than individual reflection alone. For example, one co-researcher reflected, “*It was therapeutic to stab something – there is still so much anger in me,”* illustrating how understanding emerged through action. Similarly, relational exchanges prompted shifts in perspective: “*I hadn't thought about it like that before – it made me see it differently.”* Reflections extended into everyday contexts (e.g., via Indeemo), capturing evolving and sometimes contradictory interpretations. Sense-making remained iterative rather than linear, with recovery involving the ongoing revision of meanings rather than arriving at a fixed understanding. This reframes cognition as emergent, relational practice, shaped across contexts and through collective, material, and situated forms of knowing. Rather than treating cognition as a primary driver of recovery, the framework positions it as one element in a distributed, relational practice of sense-making.

#### 3.2.4 Time

The time dimension resonates with trauma scholarship recognizing the non-linear and recursive nature of traumatic experience (9), and with Barad's (7) concept of *spacetimemattering*, which challenges linear distinctions between past, present, and future. Time in recovery was experienced as unstable and embodied, with co-researchers describing temporal dissonance in which urgency, delay, and suspension coexisted. As one reflected, “*It's always running out. Never enough and sometimes too much.”* At the same time, another noted that time “*doesn't feel like that in your body,”* highlighting how temporal experience is shaped by affect and embodiment rather than objective measure. These experiences were often at odds with institutional expectations of linear, time-bound recovery, such as the pressure to “…*be well in 24 weeks.”* Engagement with materials could also shift temporal experiences, with one co-researcher noting that “*time…seems to stand still”* when sewing. Despite these non-linear experiences, process-oriented language persisted, reflecting the enduring influence of dominant recovery narratives. Within the framework, such expressions are understood as attempts to articulate ongoing, iterative reconfigurations of living with trauma. Time also shaped when meaning-making became possible, with insights emerging only under supportive conditions such as safety, stability, and relational support. From this perspective, recovery is viewed as ‘spacetimemattering' rather than chronological progress, with change unfolding unevenly and relationally. It invites reflection on what may not yet be workable, what might now be revisited, and how service timelines support or constrain these slower conditions of recovery.

#### 3.2.5 Biological

The biological dimension resonates with research showing that trauma shapes physiological systems (e.g., stress regulation, immune function) across the life course (3), while also recognizing that these systems remain responsive to environmental and therapeutic contexts (19). Within the framework, biology is understood as one strand within broader relational, material, and social entanglements, rather than a primary determinant of recovery. Co-researchers described how trauma is lived through the body, not just remembered cognitively. As one reflected, “*your body holds the emotions… and then it pops out… in physical illness or dissociation,”* reframing physiological responses as meaningful rather than pathological. Through the research process, such experiences were increasingly understood as adaptive and regulatory. Practices such as self-soothing (“*hold myself for a moment… and just cry”*), or movement-based activities like Tai Chi, supported embodied awareness and grounding beyond verbal reflection. Encounters with healthcare systems highlighted tensions between biological and physiological interpretations, with some physical symptoms attributed solely to trauma. These accounts illustrate that biological experience cannot be separated from the relational, experiential, and material conditions through which it is lived and interpreted. This dimension, therefore, positions bodily states as active and responsive, inviting attention to how practices such as medication, movement, rest, and pacing interact with other dimensions of recovery, without reducing recovery itself to biological change. This contrasts with approaches that privilege biological regulation as the primary mechanism of recovery, instead situating physiological processes within broader relational and environmental contexts.

#### 3.2.6 Space

The space dimension highlights how environments and atmospheres shape experience and participation in trauma recovery (7, Walkerdine, 2010). Space is physical, emotional, and institutional, actively configuring what can be felt, expressed, and shared. In this study, exhibition and workshop spaces were co-created through relational and material engagement. As one co-researcher noted, “*it is our space,”* emphasizing that space is not a neutral container but produced through collective presence. Spatial arrangements, such as body maps displayed together, enabled connection and recognition, where “…* it creates a safe space, camaraderie,”* suggesting that safety emerges through interaction rather than being inherent. Importantly, space also shaped who could participate. As reflected in the simple observation that “*not everybody can speak,”* verbally focused settings may exclude some forms of expression, while more flexible environments allow alternative ways of engaging. This also highlights how homes, clinics, community venues, online forums, and policy settings can enable or constrain participation. Rather than labeling spaces as simply “safe” or “unsafe,” it invites attention to what spaces do: how they enable or constrain participation, and how they might be reconfigured to support more inclusive and relational forms of recovery.

#### 3.2.7 Relationality

The relationality dimension resonates with scholarship highlighting recovery as grounded in reconnection and the rebuilding of trust (9), while extending relationality beyond interpersonal ties to a wider ecology of human and more-than-human connections (Mascolo, 2013). Within the framework, recovery is understood as unfolding with and through others. Co-researchers emphasized the importance of shared understanding, for example, “*being with people who just get it… you don't have to explain everything,”* illustrating how recognition reduced the burden of articulation and enabled participation. Relational encounters were also generative, with experiences becoming shared and reworked collectively: “*we kind of worked through it together… it wasn't just mine anymore.”* At the same time, relationality was not uniformly positive. Moments of overwhelm (“*sometimes it felt too much…”*) highlighted how connection can both support and unsettle recovery. Such dynamics are understood as integral rather than problematic. Importantly, relationality extended beyond human interaction. Connections with materials, spaces, and environments were also described as supportive: “*it wasn't just the people… it was the space, the materials,”* suggesting *that* recovery emerges through a distributed network of relations. While relational approaches often focus on interpersonal dynamics, the framework extends relationality beyond human-to-human interaction to include material and environmental relations (animals, places, technology) as active contributors to recovery. This highlights recovery as shaped through interconnected systems of support, drawing attention to how relationships with peers, practitioners, family, community, and more-than-human matter enable or constrain what becomes possible.

#### 3.2.8 Ethics

In contrast to procedural or principle-based ethical models, the ethical dimension draws on relational and posthuman ethics to conceptualize ethics as an ongoing situated negotiation of power, responsibility, boundaries, and participation across interconnected human and more-than-human relations (Braidotti, 2013; Kimmerer, 2013). Co-researchers described tensions in which recovery was experienced as something done to them rather than with them, with decisions often shaped by institutional authority: “*it felt like decisions were already made.”* Similarly, pressures to perform “*acceptable”* forms of recovery (“*you learn what you're supposed to say”*) highlighted how certain narratives are privileged, shaping whose experiences are recognized.

Ethical questions also emerged around knowledge and its limits, particularly where recovery resisted clear articulation: “*it's not something that fits into words.”* These moments foreground the risks of imposing linear, outcome-driven models that marginalize embodied or relational forms of knowing. In contrast, co-creative practices within workshops and exhibitions fostered more reciprocal forms of engagement, where knowledge was produced collectively: “*we were part of it, not just being told.”* These spaces disrupted silencing and opened up alternative ways of expressing and witnessing experience. This dimension invites attention to how power operates across relationships, services, and environments, and how these might be reconfigured to support more equitable participation. Ethics here is understood as dynamic and relational: not eliminating power differences but making their effects visible and open to negotiation. It also extends beyond human relations, recognizing materials and environments as active participants in recovery, requiring forms of engagement grounded in care, responsiveness, and mutual influence.

## 4 Critical reflections on implications for practice, policy, and research

Taken together, RAF-T positions trauma recovery as a population health concern by recognizing that the widespread prevalence and long-term consequences of childhood trauma extend beyond individual therapeutic contexts. Rather than locating recovery solely within individual change, the framework conceptualizes it as emerging through ongoing relations among people, communities, services, institutions and the wider social and material conditions that shape health. From this perspective, RAF-T offers a conceptual basis for considering recovery across public health, community, and societal settings. This aligns with a social determinants of health perspective, shifting attention from “what works” at the individual level to how conditions enable or constrain recovery across populations and contexts.

For practitioners, RAF-T offers a heuristic framework for collaboratively exploring trauma recovery beyond symptom reduction or individual therapeutic progress. Rather than prescribing what recovery should involve or functioning as an assessment tool, it supports dialogue about how relational, biological, material, spatial, temporal, ethical, cognitive, and experiential dimensions interact and change within a person's context. This may help identify where capacities need protecting, where possibilities for growth or engagement exist, and what forms of support or environmental change might enable recovery. For example, strong engagement in therapy may coexist with unmet needs in housing, social connection, or physical health. RAF-T therefore broadens attention to the social, material, and institutional conditions of recovery, recognizing community-based and non-clinical approaches, such as peer support, creative practice, and movement-based activities, as part of a wider recovery ecosystem.

At a policy level, RAF-T has the potential to support reflection beyond checklist-style “trauma-informed” approaches toward system-level responses that attend to structural determinants of health. It highlights how conditions related to housing, welfare, service access, workforce capacity, poverty, racism, gender-based violence, and social exclusion may shape opportunities for recovery and participation across the life course. For commissioners and service leaders, RAF-T may provide a way of exploring how resources, opportunities, and barriers are distributed across interconnected services and systems, and where changes in provision or service configuration might better support recovery. It may also broaden consideration of what constitutes recovery support, including the contribution of community-based and non-clinical provision alongside clinical care. This aligns with public health priorities around health equity, prevention, and cross-sector collaboration, emphasizing that the RAF-T dimensions are situated within wider social, economic, material, institutional, and political conditions.

For research, RAF-T provides a conceptual starting point rather than a completed explanatory model. Future work should examine how different configurations of recovery emerge across populations and contexts, while refining or extending the framework through further empirical and participatory inquiry. Such questions lend themselves to interdisciplinary and mixed-methods approaches capable of examining recovery as dynamic and context-dependent. This may include realist, longitudinal, participatory and implementation-focused research exploring how different configurations of the dimensions emerge across settings, for whom, and under what conditions. Research should also examine the operationalisation of RAF-T, including its potential use in collaborative reflection, service development, care planning, and systems learning across clinical, community, and public health settings.

Despite these implications, the framework must be interpreted with several limitations in mind: RAF-T was developed within a single participatory study involving ten adult survivors of childhood trauma in a UK community context. Its dimensions are therefore provisional and context-dependent, reflecting the specific social, cultural, and material conditions through which the original inquiry was conducted. Its applicability across different populations, cultural contexts, socioeconomic conditions, trauma experiences, and health and service systems, requires further exploration.

As an emergent conceptual framework developed through participatory, post-qualitative, and arts-based inquiry, RAF-T has not yet been empirically examined beyond the context from which it was developed or evaluated in implementation. It is therefore presented as a heuristic resource for generating and organizing understanding rather than as a prescriptive or measurement-based framework. Further research should examine its applicability and utility across different settings, including how RAF-T might be operationalised in practice and how its use and contribution might be evaluated.

While RAF-T explicitly recognizes structural determinants, including poverty, housing insecurity, racism, gender-based violence, social exclusion, and wider inequalities, further empirical and conceptual work is needed to examine their relations with and across the dimensions in different contexts and populations. Translational work will similarly be required to develop and evaluate co-designed practice tools, training resources, and implementation strategies for clinical, community, and public health settings.

## 5 Conclusion

Childhood trauma is widespread, enduring, and closely linked to social, economic, and structural determinants of health. However, recovery has often been conceptualized through linear, symptom-focused models that prioritize measurement and individual regulation, with less attention to the relational, environmental, and systemic conditions that shape outcomes. In this paper, we have argued for a broader public health lens, one that recognizes how bodies, spaces, time, relationships, material conditions, and ethical dynamics interact to influence how people live with and respond to trauma. The Recovery Assemblage Framework for Trauma (RAF-T) addresses this gap by conceptualizing recovery as dynamic, relational, and contextually embedded.

Rather than positioning recovery as symptom remission or a return to a pre-trauma state, the framework conceptualizes it as an ongoing reconfiguration of relational, biological, material, spatial, temporal, ethical, cognitive, and experiential dimensions. Grounded in interdisciplinary perspectives, RAF-T articulates eight interconnected dimensions through which possibilities for recovery may be enabled, constrained, and reshaped across contexts. In doing so, it offers a potential shared conceptual language for understanding recovery as a relational and contextually embedded population health concern, shaped not only by clinical care but also by social environments, service systems, and broader inequalities.

Importantly, RAF-T is not prescriptive. Its potential value lies in its use as a reflective framework through which survivors, practitioners, policymakers, and researchers might explore what is missing, and where possibilities for change may exist. This aligns with public health priorities around health equity, systems thinking, and co-produced approaches, emphasizing recovery as collective, context-dependent, and shaped by access to resources and opportunities. RAF-T is therefore offered as a flexible and evolving framework, whose applicability and adaptation across settings and populations require further empirical examination. From a public health perspective, trauma recovery is not a fixed outcome, but is shaped through ongoing and uneven configurations of relationships, environments, material conditions, and power. Understanding recovery in this way may contribute to the development of more integrated, equitable, and context-sensitive approaches to trauma across health systems and communities.

## Footnotes

1 Indeemo is a digital platform used to collect in-the-moment reflections through text, images, and video.

2 Intra-action (Barad, 2007) refers to the idea that entities do not pre-exist their relations but emerge through them.
